# MASLD versus MAFLD in lean steatotic liver disease: diagnostic overlap, inclusivity, and the metabolically healthy lean phenotype

**DOI:** 10.1007/s10238-025-01983-7

**Published:** 2025-12-23

**Authors:** Maha Elsabaawy, Heba Demerdash, Amr Ragab, Madiha Naguib

**Affiliations:** Department of Hepatology and Gastroenterology, National Liver Institute, Menoufia University, Shebeen Elkoom, Menoufia, Egypt

**Keywords:** Lean NAFLD, MASLD, MAFLD, Metabolic health, Steatotic liver disease, Hepatic fibrosis

## Abstract

Lean non-alcoholic fatty liver disease (NAFLD) presents a unique clinical phenotype increasingly recognized within the evolving nomenclature of metabolic dysfunction–associated fatty liver disease (MAFLD) and metabolic dysfunction–associated steatotic liver disease (MASLD). This study evaluated the diagnostic applicability of both frameworks in lean Egyptian patients and explored their capacity to encompass metabolically healthy lean phenotypes. Ninety ultrasonography confirmed lean NAFLD patients (BMI < 25 kg/m²) were reassessed using MAFLD and MASLD criteria. Clinical, metabolic, and biochemical parameters, alongside non-invasive fibrosis indices and transient elastography, were compared across classifications. Among all patients, 87.8% met MASLD and 77.8% fulfilled MAFLD criteria, showing substantial overlap yet minor classification differences. MASLD identified a slightly higher proportion of lean cases, whereas MAFLD more stringently captured those with overt metabolic dysfunction. Clinical, biochemical, and fibrosis parameters were comparable among groups (*p* > 0.05). However, 12.2% of patients remained unclassified under either definition, representing a metabolically healthy lean with steatotic liver disease (MHL-SLD) subgroup. In lean steatotic liver disease, MASLD and MAFLD show substantial diagnostic overlaps and identify similar patient profiles with no significant metabolic or fibrosis differences. MASLD includes slightly more patients, yet without clear clinical distinction. Refinement of criteria may be needed to better capture metabolically healthy lean individuals with liver steatosis (MHL-SLD).

## Introduction

Fatty liver disease (FLD) represents a growing global health concern, particularly in regions such as the Middle East and North Africa, where prevalence rates exceed 50% of the adult population [1]. In Egypt, the burden of FLD continues to rise due to lifestyle transitions and high rates of metabolic comorbidities, underscoring the urgent need for early detection and accessible risk stratification tools [2].

Non-alcoholic fatty liver disease (NAFLD), first introduced in 1986, encompasses a histological spectrum ranging from simple steatosis to non-alcoholic steatohepatitis (NASH), fibrosis, cirrhosis, and hepatocellular carcinoma [3]. Although NAFLD has traditionally been associated with obesity, type 2 diabetes mellitus, and metabolic syndrome, recent data indicate that a significant proportion of patients present with normal body mass index (BMI), giving rise to the distinct phenotype of lean NAFLD [4]. This subgroup, estimated to comprise 5–20% of NAFLD cases, poses diagnostic and therapeutic challenges due to the subtlety of metabolic derangements despite a normal weight profile [5].

To reflect the central role of metabolic dysfunction in the disease’s pathogenesis, the term metabolic dysfunction-associated fatty liver disease (MAFLD) was proposed in 2020, replacing NAFLD [6]. In 2023, an international consensus further refined the terminology to metabolic dysfunction-associated steatotic liver disease (MASLD) to harmonize definitions globally and emphasize the unifying feature of hepatic steatosis [7]. Both nomenclatures aim to reduce stigma, standardize clinical criteria, and improve patient stratification across diverse populations [8].

However, the applicability of these new frameworks among lean individuals remains uncertain. Unlike obese NAFLD patients, lean individuals may develop steatosis in the absence of overt metabolic abnormalities, possibly due to genetic, epigenetic, or environmental factors [9]–[10]. This raises the question of whether the MAFLD and MASLD criteria adequately capture this subgroup or risk excluding metabolically healthy lean patients with steatotic liver disease (SLD).

The current study addresses this gap by assessing the diagnostic performance of MAFLD and MASLD criteria in a cohort of lean Egyptian patients with NAFLD. By comparing clinical, metabolic, and fibrosis profiles across classifications, the study aims to clarify the extent of overlap between these definitions and to determine which framework offers greater diagnostic validity and clinical utility in lean populations.

## Patients and methods

### Study design and population

This cross-sectional analytical study was conducted at the Hepatology and Gastroenterology Department, National Liver Institute (NLI), Menoufia University, Cairo, Egypt, between January 2023 and March 2024. A total of 90 adult patients (aged 18–65 years) with ultrasonographically confirmed NAFLD were consecutively enrolled.

The sample size (*n* = 90) was estimated using G*Power version 3.1, based on a medium effect size (Cohen’s *f* = 0.25), an alpha error of 0.05, and a power of 0.80 for comparing three diagnostic categories (NAFLD, MAFLD, MASLD). This number was increased by 10% to account for possible data loss or exclusions, yielding a final target of 90 participants.

Lean NAFLD was defined as hepatic steatosis in individuals with a body mass index (BMI) < 25 kg/m², according to the World Health Organization (WHO) criteria [11]. Patients with significant alcohol intake (> 20 g/day for men, > 10 g/day for women), viral hepatitis (HBsAg or anti-HCV positive), autoimmune hepatitis, hemochromatosis, Wilson’s disease, or hepatotoxic medication use were excluded.

### Clinical and anthropometric assessment

All participants underwent detailed clinical evaluations including history of hypertension, diabetes mellitus (DM), dyslipidemia, and medication use. Weight and height were measured using a calibrated digital scale and stadiometer, respectively, and BMI was calculated as weight (kg) divided by height squared (m²).

Waist circumference was measured at the midpoint between the lower margin of the last rib and the iliac crest, and waist-to-hip ratio (WHR) was computed. Blood pressure was measured in the seated position after five minutes of rest.

### Laboratory evaluation

Venous blood samples were obtained after an overnight fast of 10–12 h. Biochemical analyses included fasting plasma glucose, insulin, total cholesterol, triglycerides, HDL-C, LDL-C, alanine aminotransferase (ALT), aspartate aminotransferase (AST), and serum albumin.

Insulin resistance was calculated using the Homeostatic Model Assessment (HOMA-IR):

$$\begin{aligned} \frac{{{\text{Fasting}}\:{\text{Insulin}}\:(\mu {\text{U/mL}}) \times \:{\text{Fasting}}\:{\text{Glucose}}\:({\text{mmol/L}})}}{{22.5}}\end{aligned}$$

[12].

### Definition of MAFLD and MASLD

In accordance with the 2025 APASL guidelines, the diagnosis of MAFLD is based on the detection of liver steatosis (liver histology, non-invasive biomarkers or imaging) together with the presence of at least one of three criteria that include overweight or obesity, T2DM, or clinical evidence of metabolic dysfunction in lean subjects (≥ 2 metabolic abnormalities including elevated waist circumference, blood pressure ≥ 130/85 mmHg, triglycerides ≥ 150 mg/dL, low HDL-C, prediabetes, HOMA-IR ≥ 2.5, or C-reactive protein > 2 mg/L) [4].

MASLD was defined as hepatic steatosis associated with at least one cardiometabolic risk factor, in accordance with the 2023 Delphi consensus [5]. Patients with excessive alcohol consumption or other chronic liver diseases were excluded from both frameworks.

A unique group was sorted as metabolically healthy lean with steatotic liver disease (MHL-SLD), was defined by presence of SLD in lean patients with absence of all metabolic dysfunctions defined by MASLD/MAFLD criteria [5].

### Noninvasive fibrosis assessment

Liver stiffness was measured using transient elastography (FibroScan^®^, Echosens, Paris, France) after a minimum 3-hour fast. The M probe was used for all participants, and the median of 10 valid readings was recorded. Significant fibrosis was defined as ≥ 7.9 kPa (≥ F2) [13].

Two noninvasive fibrosis indices were also calculated:

- NAFLD Fibrosis Score (NFS) [14].
- FIB-4 Index [15].
- Both indices were computed automatically using standard formulae.

### Statistical analysis

Data were analyzed using IBM SPSS Statistics v28 (IBM Corp., Armonk, NY, USA). Normality of distribution was assessed using the Shapiro–Wilk test. Continuous variables were presented as mean ± standard deviation (SD) or median (interquartile range), and categorical variables as frequencies and percentages. Comparisons among MAFLD, MASLD, and NAFLD groups were conducted using one-way ANOVA or the Kruskal–Wallis test for continuous data and χ² or Fisher’s exact test for categorical data. Post-hoc analysis was performed when appropriate. Correlations were assessed using Pearson’s or Spearman’s coefficients. A *p*-value < 0.05 was considered statistically significant.

### Ethical considerations

The study protocol was approved by the Institutional Review Board of NLI. Written informed consent was obtained from all participants prior to enrollment. The study adhered to the ethical principles outlined in the Declaration of Helsinki (2013 revision).

## Results

**Fig. 1:**
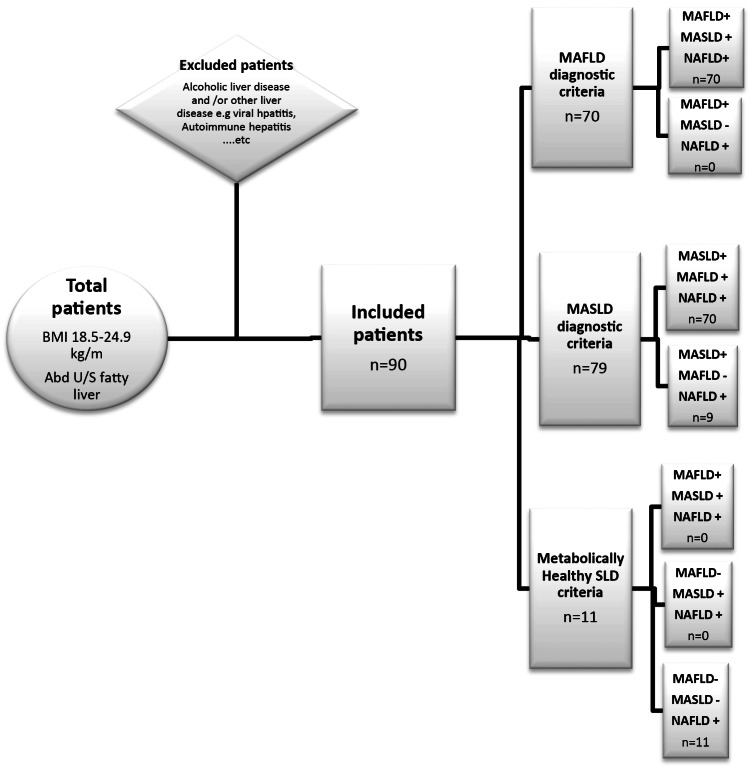
Flow chart for the new diagnostic criteria of Lean NAFLD

The present study evaluated 90 lean individuals with ultrasonographically confirmed hepatic steatosis (BMI < 25 kg/m²), with a median age of 37.5 years and a slight male predominance (56.7%) (Table 1). Overall, 87.8% (*n* = 79) met MASLD criteria and 77.8% (*n* = 70) met MAFLD criteria, demonstrating a high diagnostic concordance between the two frameworks. Only 12.2% (*n* = 11) of patients were unclassified by either definition, constituting the metabolically healthy lean steatotic liver disease (MHL-SLD) subgroup (Fig. 1).

**Fig. 2:**
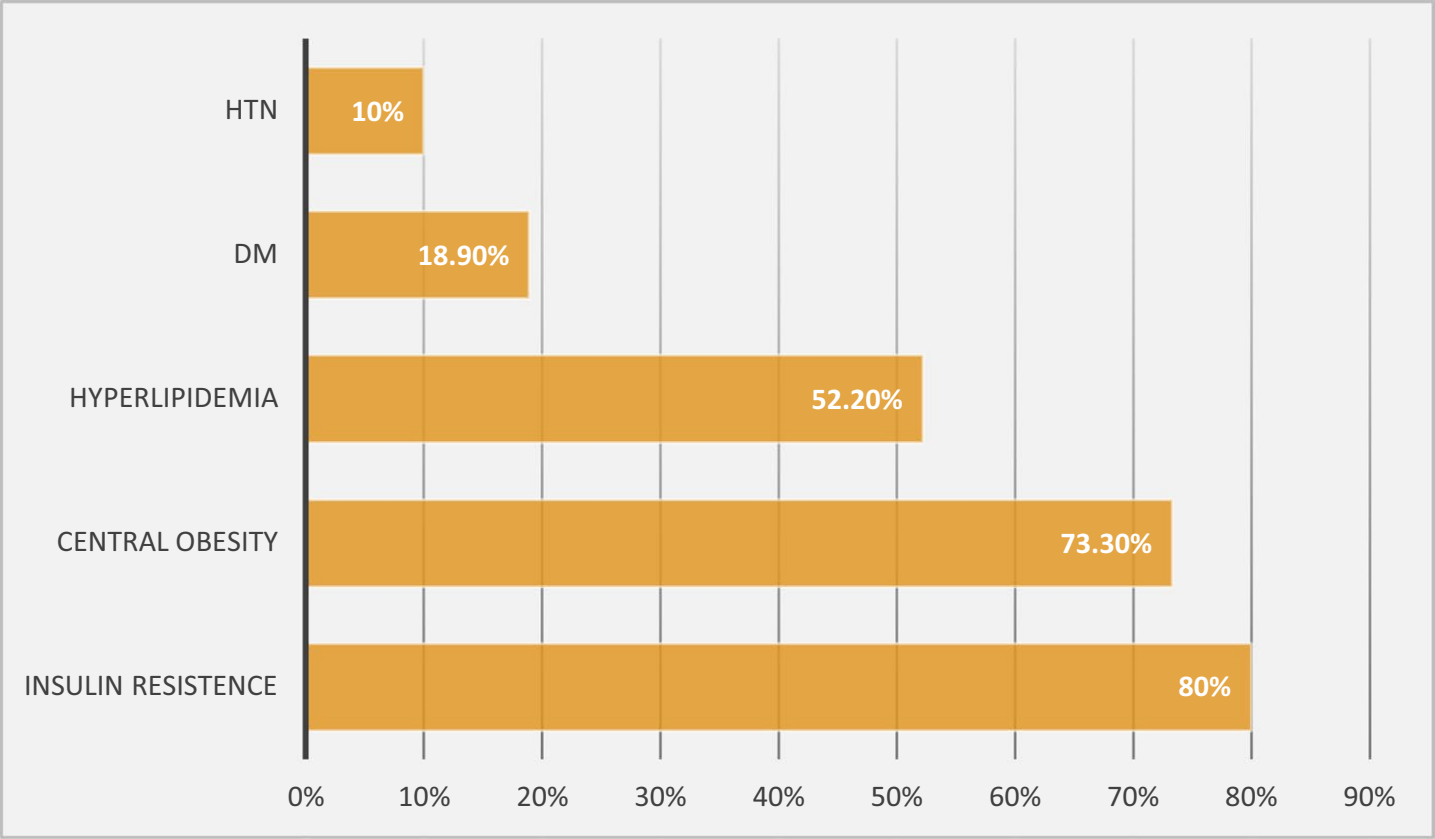
Cardio-metabolic risk factors in lean NAFLD patients

Figure 2 illustrates the comparable distribution of cardiometabolic risk factors among diagnostic categories, reinforcing that MASLD’s marginally broader inclusivity does not translate into measurable metabolic or fibrotic distinction.

Across all three classifications—NAFLD, MASLD, and MAFLD—demographic, anthropometric, and biochemical parameters were comparable (Table 1). The median BMI remained consistent at 24.5 kg/m² (*p* = 0.862), and no significant differences were observed in liver enzymes (ALT, AST), bilirubin, or albumin levels (*p* > 0.05). The distribution of sex, age, hypertension, and diabetes was also similar (*p* = 0.471, 0.935, 0.949, and 0.664, respectively), underscoring that both MASLD and MAFLD identify nearly identical lean populations with steatotic liver disease.

Metabolic risk profiling (Table 2) revealed insulin resistance as the most prevalent feature, detected in approximately 78–80% of all patients (HOMA-IR ≥ 2.5). Central obesity was present in 84–87%, while dyslipidemia affected roughly 50–52%. Hypertension and diabetes were less common, each occurring in about 10–12% of cases. The similarity of these frequencies across all groups further supports diagnostic equivalence between MASLD and MAFLD definitions in lean individuals.

Fibrosis assessment by transient elastography demonstrated low fibrosis burden, with 8.9–10% of participants showing stiffness values ≥ F2 (Table 3). Non-invasive fibrosis indices—FIB-4 (median ≈ 0.7), NAFLD fibrosis score (median ≈ − 2.8), and APRI (median ≈ 0.3)—did not differ significantly among groups (*p* > 0.05), indicating comparable liver injury profiles. Similarly, cardiovascular risk assessment using ASCVD scores (Table 4) revealed predominantly low-risk status (> 85% low risk, *p* = 0.868), with no meaningful difference among classifications.

The MHL-SLD subgroup (*n* = 11) (Table 5) was exclusively males, lean (mean BMI 24.47 ± 0.41 kg/m²), and metabolically healthy—none had diabetes, hypertension, or dyslipidemia. HOMA-IR was 3.15, fasting glucose 98.98 mg/dL, and HbA1c 5.2%, reflecting mild insulin resistance despite preserved glycemic control. Liver enzymes were modestly elevated (median ALT 35 U/L, AST 27.5 U/L), and fibrosis indices (FIB-4 = 0.69, NFS = − 3.89) confirmed minimal hepatic injury. These findings highlight a distinct lean phenotype excluded by both MASLD (as elevated HOMA-IR is not included in MASLD criteria) and MAFLD (as MAFLD criteria needs two metabolic factors and here we have only elevated HOMA-IR), yet demonstrating steatosis of potential clinical relevance.

**Table 1:** Comparison between the studied groups according to different parameters

	NAFLD (*n* = 90)	MASLD (*n* = 79)	MAFLD (*n* = 70)	Test of Sig.	*P*
Female Gender	39 (43.3%)	39 (49.4%)	37 (52.9%)	χ^2^ = 1.504	0.471
Age (years)	37.50 (33.0–42.0)	38.0 (33.0–42.0)	37.0 (33.0–41.0)	H = 0.134	0.935
BMI (kg/m ^2^ )	24.5 (24.0–24.8)	24.5 (24.0–24.8)	24.5 (24.2–24.8)	H = 0.298	0.862
AST	35.0 (25.0–46.0)	36.0 (27.0–46.0)	35.0 (26.0–46.0)	H = 0.107	0.948
ALT	38.0 (30.0–44.0)	38.0 (31.0–45.0)	38.50 (35.0–45.0)	H = 1.330	0.514
Total Bilirubin	0.90 (0.70–1.0)	0.90 (0.70–1.0)	0.90 (0.70–1.0)	H = 0.094	0.954
Alk-ph	88.0 (73.0–106.0)	84.0 (72.5–104.0)	79.5 (72.0–100.0)	H = 1.597	0.450
GGT	37.0 (28.0–45.0)	37.0(27.50–44.50)	36.0 (28.0–44.0)	H = 0.350	0.839
PC	100 (96–100)	100 (96–100)	100 (96–100)	H = 0.190	0.909
INR	1.007 ± 0.052	1.009 ± 0.053	1.008 ± 0.055	F = 0.018	0.982
Albumin	4.27 ± 0.27	4.28 ± 0.28	4.29 ± 0.27	F = 0.113	0.894
S. Creatinine	0.90 (0.70–1)	0.88 (0.70–0.98)	0.87 (0.70–0.98)	H = 0.323	0.851
Hb	12.5 (11.9–14)	12.50 (12–14)	12.55 (12–14)	H = 0.054	0.974
WBCs	7.10 (5.20–8)	7.10 (5.20–85)	7.10 (5.30–8.20)	H = 0.126	0.939
Platelet	244 (193–287)	239 (193–283.50)	244.5 (200–287)	H = 0.327	0.849

Normally distributed data was expressed using Mean ± SD. While not normally distributed data was expressed using Median (IQR) IQR: Inter quartile range SD: Standard deviation

F: F for One way ANOVA test H: H for Kruskal Wallis test χ^2^: Chi square test

p: p value for comparing between the three studied groups

*: Statistically significant at *p* ≤ 0.05

**Table 2:** Comparison between the studied groups according to different cardiometabolic risk factors

	NAFLD (*n* = 90)	MASLD (*n* = 79)	MAFLD (*n* = 70)	Test of Sig.	*P*
HTN	10 (11.1%)	10 (12.7%)	8 (11.4%)	χ^2^ = 0.105	0.949
DM	17 (18.9%)	17 (21.5%)	11 (15.7%)	χ^2^ = 0.819	0.664
BMI (kg/m ^2^ )	24.5 (24.0–24.8)	24.5 (24.0–24.8)	24.5 (24.2–24.8)	H = 0.298	0.862
T. cholesterol	160 (153–230)	160 (153.50–233)	159 (153–234)	H = 0.086	0.958
HDL	45 (41–49)	45 (41–48.50)	44 (41–48)	H = 0.534	0.766
LDL	98.4 (90.8–131.0)	98.40 (91.50–143)	97.2 (90.8–154.7)	H = 0.148	0.929
Triglycerides	102.5 (97–130)	103 (97–142.50)	102 (96–140)	H = 0.158	0.924
FBS	100.3 (92–112)	100 (91.13–113)	100 (91.27–112.1)	H = 0.021	0.990
HbA1C	5.10 (4.90–5.60)	5.10 (4.90–5.65)	5 (4.90–5.50)	H = 0.649	0.723
Waist circumference	104.4 (99.0–115.0)	108.2 (99.7–116.95)	109.3 (100.0–117.0)	H = 1.056	0.590
Hip circumference	106.4 ± 13.83	106.9 ± 14.27	107.5 ± 14.49	F = 0.118	0.889
Waist/Hip	0.988 ± 0.084	0.990 ± 0.084	0.992 ± 0.084	F = 0.062	0.940
Fasting insulin	12.15 (9.0–15.0)	12.0 (9.0–15.0)	12.20 (9.0–15.20)	H = 0.889	0.641
HOMA IR	3.10 (2.62–3.64)	3.10 (2.58–3.62)	3.15 (2.64–3.70)	H = 0.729	0.695

**Table 3:** Assessment of hepatic fibrosis in the studied groups

	NAFLD (*n* = 90)	MASLD (*n* = 79)	MAFLD (*n* = 70)	Test of Sig.	*P*
APRI	0.28 (−1.10–0.58)	0.33 (−0.65–0.58)	0.28 (−1.12–0.42)	H = 0.451	0.798
FIB-4	0.74 (0.57–1.22)	0.77 (0.61–1.19)	0.72 (0.61–1.13)	H = 0.408	0.815
HSI score	34.26 ± 3.89	34.30 ± 4.06	34.82 ± 3.90	F = 0.464	0.629
NAFLD fibrosis score	−2.83 (−3.70 – −2.08)	−2.79 (−3.56 – −2.08)	−2.83 (−3.70 – −2.28)	H = 0.543	0.762
*Fibroscan*					
< F2	*N* = 82 (91.1%)	*N = 72 (91.1%)*	*N = 63 (90%)*		
≥ F2	*N = 8 (8.9%)*	*N = 7 (8.9%)*	*N = 7 (10%)*		

Normally distributed data was expressed using Mean ± SD. While not normally distributed data was expressed using Median (IQR) IQR: Inter quartile range SD: Standard deviation

F: F for One way ANOVA test H: H for Kruskal Wallis test χ^2^: Chi square test

p: p value for comparing between the three studied groups

*: Statistically significant at *p* ≤ 0.05

**Table 4:** Atherosclerotic-cardiovascular disease (ASCVD) assessment in the studied groups

	NAFLD (*n* = 90)	MASLD (*n* = 79)	MAFLD (*n* = 70)	Test of Sig. *P* value
ASCVD	0.02 (0.01–0.40)	0.02 (0.01–0.40)	0.02 (0.01–0.40)	H = 0.282 *P* = 0.868
*ASCVD risk*				
Low	79 (87.8%)	69 (87.3%)	64 (91.4%)	χ2 = 1.496
Intermediate	3 (3.3%)	3 (3.8%)	1 (1.4%)	
Borderline	3 (3.3%)	2 (2.5%)	2 (2.9%)	^MC^*p *= 0.982
High	5 (5.6%)	5 (6.3%)	3 (4.3%)	

**Table 5:** Characteristics of metabolically healthy lean steatotic liver disease (MHL-SLD) (*n* = 11)

	No. (%)	Min. – Max.	Mean ± SD	Median (IQR)
Male gender	11 (100%)			
DM	0 (0%)			
HTN	0 (0%)			
Age (years)		22.0–65.0	36.40 ± 11.05	37.0 (12.75)
BMI (kg/m ^2^ )		23.8–24.9	24.47 ± 0.41	24.6 (0.75)
Waist circumference (cm)		88.0–101.5	97.6 ± 5.09	99.35 (5.83)
Waist/hip		0.90–1.1	0.97 ± 0.05	0.96 (0.08)
ALT		17.0–120.0	41 ± 28.76	35.0 (13.5)
AST		11.0–113.0	35.60 ± 28.24	27.50 (13)
GGT		25.0–68.0	44.30 ± 15.68	44.0 (28.25)
Platelet		179.0–410.0	300.4 ± 79.46	287.0 (142)
T. cholesterol		147.0–277.0	183.2 ± 44.37	159.0 (64)
HDL-C		41.0–66.0	47.25 ± 7.02	45.0 (6.25)
LDL-C		81.40–175	103.34 ± 27.72	94.90 (23.8)
Triglyceride		97.0–125.0	105.4 ± 9.60	102.5 (11)
Fasting blood glucose		78.0–99.0	92.90 ± 2.44	89.98 (5.96)
Glycosylated-Hb		4.80–6.37	5.31 ± 0.47	5.2 (0.525)
HOMA-IR		2.8–3.7	3.29 ± 0.34	3.15 (0.67)
ASCVD		0.005–0.074	0.012 ± 0.022	0.005 (0.004)
APRI		−1.67–0.66	−0.51 ± 0.9	−0.45 (1.67)
FIB-4		0.24–1.30	0.69 ± 0.38	0.54 (0.70)
Hepatic steatosis index		31.45–37.77	34.34 ± 2.40	33.56 (5.17)
NAFLD fibrosis score		−5.03 – −1.86	−3.89 ± 1.09	−4.15 (−1.76)
*Fibroscan*				
F0	9(81.8%)			
F1	1 (9.1%)			
*F2*	1 (9.1%)			

## Discussion

NAFLD challenges traditional paradigms by manifesting in individuals with normal BMI but variable metabolic risk [16]. With the transition from NAFLD to MAFLD and, more recently, to MASLD, there is increasing emphasis on metabolic health as a diagnostic cornerstone [17, 18]. However, the applicability of these evolving frameworks to lean populations—particularly those with subtle or absent metabolic abnormalities—remains uncertain.

The primary objective of this study was to assess the diagnostic overlap and clinical equivalence between MASLD and MAFLD in lean individuals. Our results demonstrate substantial concordance between the two nomenclatures, identifying nearly identical patient profiles without significant differences in metabolic parameters or fibrosis indices. These findings suggest that, in lean patients, both definitions perform comparably in disease identification, supporting the concept of diagnostic equivalence rather than distinction.

Genetic and environmental factors may contribute to disease variability among lean individuals with steatotic liver disease. Variants such as *PNPLA3* and *TM6SF2* have been associated with hepatic fat accumulation independent of metabolic syndrome, while lifestyle and environmental exposures—including diet composition, physical inactivity, and gut microbiome dysbiosis—may further influence disease expression [9, 10]. These factors could partly explain the observed heterogeneity within lean MASLD and MAFLD phenotypes.

Although MASLD identified a marginally higher number of lean patients, this difference was not accompanied by statistically or clinically significant variations in metabolic profile or fibrosis stage, indicating diagnostic equivalence rather than superiority.

Despite this overlap, a small but clinically relevant subset of our patients (≈ 13%) remained unclassified under both models. These metabolically healthy lean steatotic liver disease (MHL-SLD) individuals had normal glucose and lipid profiles yet demonstrated hepatic steatosis and mild insulin resistance. Their exclusion highlights an inherent limitation of both frameworks, particularly MASLD—which, although broader, still depends on cardiometabolic risk factors as diagnostic entry points. The MHL-SLD subgroup underscores a diagnostic blind spot in both systems, emphasizing the need for future frameworks that integrate body composition, genetic markers, and metabolic biomarkers, especially HOMA-IR to refine disease classification.

The concept of MHL-SLD aims to distinguish patients with hepatic steatosis without metabolic dysfunction or secondary causes. Unlike the term cryptogenic SLD, which implies an unidentified etiology, MHL-SLD acknowledges these individuals as part of a metabolic spectrum with potential for transition toward metabolic dysfunction over time. This reclassification may support earlier surveillance, metabolic risk assessment, and personalized counseling rather than assuming a benign or idiopathic course.

To achieve true inclusivity, the MASLD model must evolve beyond its current metabolic boundaries. Hepatic steatosis in lean individuals can arise from non-metabolic mechanisms including genetic predisposition (*PNPLA3*, *TM6SF2*), gut microbiota dysbiosis, sarcopenia, and environmental influences such as diet and hormonal regulation [9, 19]. Recognizing these drivers as legitimate diagnostic pathways would prevent the exclusion of lean patients whose disease is biologically distinct but clinically significant.

Furthermore, reliance on BMI underestimates visceral adiposity, a key determinant of hepatic lipid accumulation even in lean subjects. Incorporating waist circumference, waist–hip ratio, or imaging-derived visceral fat indices as diagnostic metrics would improve sensitivity for detecting metabolically unhealthy lean steatosis [20, 21].

In addition, MASLD should integrate molecular and functional biomarkers reflecting early metabolic stress and hepatic injury—such as hepatokines (FGF21, fetuin-A), inflammatory markers (CK-18, PRO-C3), and metabolomic signatures of insulin sensitivity [21, 22]. These markers could allow for precision stratification of lean individuals along a metabolic continuum, capturing those at risk before overt dysfunction develops.

Finally, acknowledging a formal subcategory such as MHL-SLD would ensure that individuals with hepatic steatosis but no measurable metabolic derangements are not diagnostically invisible. This approach mirrors the recognition of metabolically healthy obesity and would allow longitudinal characterization of its natural history and potential for transition to metabolically unhealthy states.

Recent studies further support the heterogeneity of lean steatotic liver disease. Wang et al. (2024) reported that lean individuals meeting MAFLD criteria exhibited subtle metabolic derangements—particularly insulin resistance and altered adipokine signaling—despite normal BMI, suggesting that conventional cardiometabolic thresholds may underestimate risk in lean populations [23]. Similarly, Katsiki et al. (2024) demonstrated that lean MASLD patients with preserved metabolic profiles experienced hepatic inflammation and fibrosis progression, highlighting that non-traditional mechanisms such as sarcopenia, visceral adiposity, “thin-outside–fat-inside” (TOFI) body composition phenotype, even in the absence of overt obesity, and genetic variants (PNPLA3, TM6SF2) contribute to disease pathogenesis [24]. These findings align with our observation of a metabolically healthy lean SLD subgroup, underscoring the diagnostic limitations of current MAFLD/MASLD criteria and the need for refined definitions incorporating metabolic biomarkers and body composition metrics.

A more inclusive MASLD model—enriched by non-metabolic determinants, body composition metrics, and biomarker-based risk stratification—would bridge the gap between population-wide screening and individualized management.

This study’s strengths include its focused evaluation of MAFLD and MASLD among lean NAFLD patients, use of transient elastography for objective fibrosis assessment, and exploration of the unclassified MHL subgroup. However, this study has several limitations that merit acknowledgment. Its single-center design, modest yet statistically powered sample size, and cross-sectional framework limit generalizability and preclude causal or longitudinal inference. Although ultrasonography was selected for its accessibility and practicality, its relatively low sensitivity for mild steatosis may have led to underestimation of disease prevalence and contributed to sampling variability between MASLD and MAFLD groups, potentially inflating the risk of type II error. In addition, the absence of genetic and inflammatory profiling constrains mechanistic interpretation and prevents deeper insight into the underlying pathophysiology. Moreover, the small number of patients classified exclusively as MASLD-only or MAFLD-only limited subgroup comparisons, precluding a full characterization of their distinct metabolic or clinical phenotypes. These limitations collectively underscore the need for larger, multicenter, and longitudinal studies utilizing advanced imaging and comprehensive biomarker panels to validate and extend the present findings.

Although this study primarily addressed the diagnostic comparability of MASLD and MAFLD, understanding their therapeutic implications is equally important. The adoption of a unified and pathophysiology-based classification could ultimately inform individualized management strategies and facilitate risk stratification in clinical trials. Future longitudinal studies are needed to determine whether these diagnostic categories predict response to pharmacologic or lifestyle interventions. Future work should also aim to validate expanded MASLD criteria across diverse ethnic populations and assess longitudinal outcomes, particularly for MHL individuals. Large-scale multicenter studies integrating genetic, metabolic, and molecular profiling will be critical to defining refined diagnostic thresholds. The development of a multidimensional, biomarker-informed “MASLD+” framework could enable a unified and inclusive classification system encompassing both metabolic and non-metabolic etiologies of steatotic liver disease.

In conclusion, MASLD and MAFLD demonstrate substantial diagnostic concordance within the lean steatotic liver disease population, identifying largely overlapping cohorts without discernible differences in metabolic or fibrosis parameters. Although MASLD encompasses a marginally broader subset of individuals, this increased inclusivity does not appear to confer distinct clinical or pathophysiological advantages. The recognition of a metabolically healthy lean SLD subgroup highlights a diagnostic gap in current frameworks. Refinement of MASLD criteria—particularly through integration of insulin resistance markers such as HOMA-IR may enhance the detection of early metabolic risk and improve disease stratification in lean populations.

## Data Availability

No datasets were generated or analysed during the current study.

## Abbreviations

NAFLD: Non-alcoholic fatty liver disease
MAFLD: Metabolic associated fatty liver disease
MASLD: Metabolic associated steatotic liver disease
SLD: Steatotic liver disease
HTN: Hypertension
DM: Diabetes mellitus
BMI: Body mass index
AST: Aspartate aminotransferase
ALT: Alanine aminotransferase
GGT: Gamma-glutamyl transferase
INR: International normalized ratio
HDL: High-density lipoprotein
LDL: Low-density lipoprotein
TG: Triglycerides
HbA1C: Hemoglobin A1c
WC: Waist circumference
W/H: Waist-to-hip ratio
HOMA-IR: Homeostasis model assessment of insulin resistance
APRI: AST-to-platelet ratio index
FIB-4: Fibrosis-4 score
NFS: NAFLD fibrosis score
M: Mean
SD: Standard Deviation
OR: Odds ratio
CI: Confidence Interval
QR: Inter quartile range
SD: Standard deviation
